# Infant and Young Child Feeding Practices and Health

**DOI:** 10.3390/nu15051184

**Published:** 2023-02-27

**Authors:** Cristiana Berti, Piotr Socha

**Affiliations:** 1 Pediatric Area, Fondazione IRCCS Ca’ Granda Ospedale Maggiore Policlinico, 20122 Milan, Italy; 2 Public Health Department, Children’s Memorial Health Institute, 04-730 Warsaw, Poland

Early childhood feeding practices are fundamental for a child’s healthy growth, development and potential. Infancy is also the proper time for shapinglife-long dietary patterns that are health-promoting for both people and the planet. The World Health Organization and the United Nations Children’s Fund have established a global strategy for optimal infant and young child feeding, including exclusive breastfeeding (EB) for the first 6 months of life and the introduction of nutritionally adequate and safe complementary foods at 6 months, along with continued breastfeeding for children up to 2 years of age or beyond [1]. EB is acknowledged to provide a balanced supply of nutrients, bioactive proteins, indigestible oligosaccharides, signaling system components and bifidogenic bacteria [2], as well as protection against infection [3]. Furthermore, breastfeeding is likely associated with lower risk of overweight and diabetes [3] and possibly has positive effects on cognition [4]. Timely, adequate, and safe complementary feeding (CF), in terms of the quantity, quality, variety, consistency and safety, is paramount to the promotion of health, support of growth and enhancement of development [1]. Dietary patterns are established between 1 and 2 years of age and track into mid-childhood, with those characterized by added sugars, unhealthy fats and poor consumption of fish being the most stable throughout childhood [5]. Moreover, some nutrients may have life-long programming effects, as has been shown for diets based on a high protein intake in infancy, increasing the risk of obesity later on [6].

However, many infants and children worldwide do not receive adequate feeding, with potentially harmful effects on nutrition and health outcomes that are perpetuatedacross generations. Based on the current figures, the world is not on course to achieve the Sustainable Development Goals required to eliminate malnutrition [7]. Moreover, due to higher intakes of minimally nutritious, ultra-processed foods, today’s children face a double burden of malnutrition [8]. Uncertainty about thefuture due to climate change, conflicts and inequalities also threatens child health and well-being [9,10].

Several factors and determinants influence infant and young child feeding and coincide to establish dietary practices. Eating habits evolve during the first years of postnatal life as biological, cognitive and environmental processes [11]. Indeed, morphological, physiological and functional adaptations allow the child to manage changes in their diet, moving from milk to an increasing variety of foods. Infants and toddlers learn to enjoyfood flavors and textures as a consequence of repeated exposure to a variety of foods in early life; the caregiver’s responsiveness to their hunger cues and emotional states; and the caregiver’s beliefs and attitudes regarding childnurture. The relationship established with food also depends on family characteristics, nutritional knowledge, social influences, cultural customs, and food contexts. Certainly, the quality of dietary patterns and the related nutrition outcomes are not simply a matter of personal choice. Making healthy choices is difficult for socially, educationally, economically or politically disadvantaged members of the population [12]. Feeding problems in children, which havebeen more commonly recognized in recent years, can originate from poor parent–child interactions in regard to responsive feeding [13].

To improve dietary patterns and health status during the lifecourse, it is important to understand the factors underlying the regulation of food intake and habits at this age. In particular, further research is needed to address the current knowledge gaps regarding the long-lasting influences of qualitative and/or quantitative differences in early dietary intakes and interactions. Furthermore, there is the need to investigate and tackle challenges and barriers (i.e., climate change; food prizes; socioeconomic inequities) which affect everyday circumstances and environments (i.e., food accessibility and affordability; provision for infant care; healthcare environments), delaying progress in efforts to fight malnutrition. A more holistic view of health and nutrition is paramount. Through-the-life-course nutrition education and research, integrated into multisector programs and starting at an early age, may have important implications for long-term health.

This Special Issue gathers recent high-quality research articles in the field of breastfeeding, weaning and infant and young child health in anattempt to improve our knowledge about these important topics.

The review by Agostoni and colleagues [14] discusses the key issues related to child malnutrition with a particular focus on feeding practices, climate change, the COVID-19 pandemic and food systems. Moreover, it provides suggestions to improve children’s lives and futuresfromthe perspective of sustainable nutrition in early life as a novel approach to the fight against malnutrition.

Huss et al. [15] carried out two studies aiming to assess the nutritional compositions, including the carbohydrate profiles, of home-prepared versus commercially prepared fruit and vegetable infant purees. In the commercially prepared purees, 88% of micronutrients were retained compared to the homemade ones. In both types, more than 90% of the carbohydrate fraction consisted of free sugars, and the estimated glycemic load was low. These results suggest that both preparations are nutritious and appropriate options for infants’ diets.

The study conducted by Liao et al. [16] is the first to investigate the relationship between the serum zinc level and several Toll-like receptor-triggered cytokine responses and among the few studies exploring the role of zinc, focusing on a prospective cohort of Taiwanese zinc-sufficient children. Despite an adequate serum concentration, zinc status was still associated with an altered innate cytokine response and infectious outcomes, highlighting the importance ofensuring a proper intake of zinc-rich foods in children’s daily diets.

In order to identify priority areas to be targeted through appropriate policies and interventions aiming to improve child nutrition in Uganda, Scarpa et al. [17] analyzed the 2016 Ugandan Demographic Health Survey dataset, including 5485 children aged 6–23 months. Albeit that it has undergone some improvements, it was found that the CF practice remains suboptimal, with the northern districts showing a low percentage of achievement of CF indicators among children. Health status, vaccination status and wealth were confirmed as predictors of minimalmeal frequency and minimaldietary diversity. 

The retrospective cohort study conducted by Huang and colleagues [18] explored the effects of breastfeeding for the first 4 months of life on thinness, overweight and obesity in children aged 3 to 6 years in Eastern China and analyzed the influential factors. The findings of this work support the notion that breastfeeding is protective against overweight or overweight/obesity.

Ali et al. [19] conducted a survey to address research gaps on feeding practices and dietary intake among infants and toddlers in the northern regions of the United Arab Emirates, finding that less than 50% of children were exclusively breastfed, and the frequency of the intake of nutrient-dense food groups was sub-optimal. These results underline the need to providemothers with information on healthy feeding practices.

The cross-sectional study conducted by Mantzorou and colleagues [20], including 2515 mother–child pairsfrom nine Greek rural and urban regions, found that EB for at least 4 months has a protective role againstboth postpartum maternal weight loss and childhood overweight and obesity.

Faber et al. [21] investigated the feasibility of daily egg consumption among infants aged 6 to 9 months in terms of dietary intake, the usage of eggs, allergy symptoms and the perceived effects of lockdown on child feeding and care in a peri-urban area in South Africa, showing that frequent egg consumption may contribute safely to CF.

To determine whether nutrient adequacy and dietary diversity among 6–24-month-old South African children were associated with the cost of the diet, Mulabisano et al. [22] analyzed data of 24 h dietary recalls from independent studies and retail food prices. The observed higher costs of more nutritious diets highlight the value of considering affordability in interventions aiming improve diet quality among children under 2 years of age.

Mulville et al. [23] carried out the first study on caregivers’ CF experience, including transgenerational practices, in Native Hawaiian and Other Pacific Islander and Filipino populations. The caregivers practicedcaution in making CF decisions and followed the advice of healthcare professionals (HCPs), albeit that this was conflicting, regarding the timing of CF introduction instead of relying on their families’ advice. These data confirm the importance of HCPs in providing consistent CF counseling.

Based on observations of 67 mothers and their 6–18-month-old children in Bangladesh, Black and colleagues [24] laid the groundwork for the development of a valid and reliable measure of responsive feeding by incorporating bidirectional mother–infant responsivity and early learning principles into the measurement and validation. This work supports responsive feeding as a tool for modulating between proximal and distal responsivity and enabling children to acquire and practice healthy eating behaviors.

The work conducted by Masztalerz-Kozubek et al. [25] revealed that early feeding factors such as the breastfeeding duration, types of complementary foods and mealtime environment in the first months of CF may be linked to eating behaviors (i.e., enjoyment of food, desire to drink, satiety responsiveness and slowness in eating) among children aged 1–3 years. In light of this, the authors recommend that parents of toddlers should receive advice about the importance of breastfeeding, responsive feeding and mealtime environments.

In their quasi-experimental intervention design study conducted in Taiwan, Huang and colleagues [26] evaluated the impacts of different mother–infant skin-to-skin contact (SSC) regimens on newborn breastfeeding outcomes. Based on their results, the authors advise healthcare personnel to provide early SSC for at least 1 h to improve breastfeeding success and to promote health benefits for the mother–child pair.

Using data of a national cohort of children from low-income families, Thomson et al. [27] explored the relationship between breastfeeding and initial vegetable introduction and vegetable intake in early childhood. They found that longer breastfeeding and introduction to a greater variety of vegetables at 9 months can lead to an increase in the consumption of a greater variety of vegetables by young children.

Comparing children attending Guatemala City Municipal Nurseries (GCMN) versus matched controls, Palacios et al. [28] observed that the GCMN children had greater math and fluid intelligence scores and lower odds of stunting. These findings support the importance of integrating early childhood nutrition and education interventions into methods for improving cognitive and growth outcomes to reduce health disparities in low-resource settings using childcare institutions.

Through their detailed literature search focusing on the nutrition settingsof children aged under 5 years in the Eastern Mediterranean Region (EMR), Ibrahim and colleagues [29] found evidence of suboptimal infant and young child feeding patterns and a twofold-increased incidence of malnutrition. Hence, the authors advocate the prioritization of measures designed to improve children’s nutrition in the EMR.

The review by Zama et al. [30] provides an up-to-date overview on the association between the gut microbiota (GM) and respiratory tract infections (RTIs) in children. Despitemounting evidence of GM dysbiosis, no distinct bacterial signature associated with RTI predisposition was identified to suggest that further research in this field is needed.

As guidelines and practices related to nutrition for children aged 12–24 months vary substantially across countries, the perspective paper of Reverri and colleagues [31] compared differences in the US and globally to identify knowledge and surveillance gaps that need to be filled across the spectrum of young child feeding in order to support the nutrition and health of this population.

Taken together, the findings ofthe papers included in this Special Issue may act as abasis for future research on early childhood feeding practices and provide policy-makers, practitioners and academics with suggestions for preventive nutrition-based strategies so as to break the intergenerational cycle of malnutrition and adverse health outcomes.
